# Enrichment, Isolation and Characterization of Heavy Metal-Tolerant Bacteria from Polar Lacustrine Sediments

**DOI:** 10.3390/microorganisms13020389

**Published:** 2025-02-10

**Authors:** Alessandro C. Rappazzo, Alessia Marchetta, Carmen Rizzo, Maurizio Azzaro, Warren R. L. Cairns, Angelina Lo Giudice, Maria Papale

**Affiliations:** 1 Institute of Polar Sciences, National Research Council, 98122 Messina, Italy; alessandrociro.rappazzo@isp.cnr.it (A.C.R.); carmen.rizzo@szn.it (C.R.); maurizio.azzaro@cnr.it (M.A.); maria.papale@cnr.it (M.P.); 2 Department of Environmental Sciences, Informatics and Statistics, Ca’ Foscari University of Venice, 30172 Mestre, Italy; warrenraymondlee.cairns@cnr.it; 3 Department of Chemical, Biological, Pharmaceutical and Environmental Sciences, University of Messina, 98166 Messina, Italy; alessia.marchetta@unime.it; 4 Stazione Zoologica Anton Dohrn, Sicily Marine Centre, Department Ecosustainable Marine Biotechnology, 98167 Messina, Italy; 5 Institute of Polar Sciences, National Research Council, 30172 Venice, Italy; 6 Italian Collection of Antarctic Bacteria of the National Antarctic Museum (CIBAN-MNA), 98166 Messina, Italy

**Keywords:** heavy metal tolerance, *Pseudomonas*, lacustrine sediments, bacterial isolates, biosequestration

## Abstract

Polar areas are not exempt from anthropogenic pollution. Heavy metals have been detected in Arctic and Antarctic lakes. Bacteria, at the base of the food web, can possess the ability to adsorb or immobilize heavy metals in the environment and reduce their concentration in the water column. However, several gaps exist in our knowledge of bacterial tolerance to heavy metals in polar systems, especially in lakes. Heavy metal-tolerant bacteria from polar lacustrine sediments were selectively enriched and subsequently isolated and identified. Their growth at increasing concentrations of different heavy metals (iron, copper, and mercury) was evaluated. Selected isolates were tested for sequestration of iron and mercury. A total of 101 bacterial isolates were obtained from metal-enriched cultures. Gammaproteobacteria and Actinomycetota isolates were most abundant in Arctic and Antarctic enrichments, respectively. Iron was the most tolerated metal. Mercury and iron were sequestered by the isolates by up to 14.2 and 13.4%, respectively. The results from this study contribute to our understanding of heavy metal-tolerant bacteria from cold environments and their potential use in biotechnological applications.

## 1. Introduction

The widespread perception is that Polar Regions are pristine. Nevertheless, by a process of long-range transport (across national borders at varying rates, e.g., through pathways like water or air), they can be reached by a plethora of contaminants originating from thousands of miles away, thus becoming a sink and potential reservoir of legacy pollutants [1,2,3]. Lakes lying in extremely remote areas, such as the High Arctic and Antarctica, are sensitive reference systems for measuring human impacts such as global climate change and long-range pollution transport. Despite the adoption of precautions, they have become increasingly affected by airborne long-range and locally emitted (e.g., from research activities and tourism) organic and heavy metal contaminants [4,5]. In the Arctic, military installations, industrial emissions, mining activities, small settlements, power generators, and vehicles are all local sources of pollution [6]. In aquatic environments, lake sediments act as a sink for metals, where they can accumulate at concentrations higher than those in the overlying waters [7]. Several investigators have demonstrated the occurrence of heavy metals in the sediments of Arctic [3,8,9,10] and Antarctic [11] lacustrine systems. Polar lakes are dominated by the microbial loop, a model that describes the cycling of carbon and nutrients through microorganisms, including viruses, bacteria, phytoplankton, zooplankton, and protozoa [12], in aquatic environments. Among these, bacteria are the most prevalent sedimentary organisms, representing the first step in the transfer of toxic compounds, including metals, to higher trophic levels. By means of their genetic and biochemical capabilities, bacteria can adopt strategies (via metabolic pathways or resistance mechanisms) allowing them to adsorb, accumulate, and transform heavy metals in most food chains [13], with implications for their repurposing in bioremediation. A number of metals are essential micronutrients for microbial growth (e.g., zinc, copper, nickel, chrome, iron) and are involved in cellular processes such as redox reactions, as coenzymes, and in osmotic regulation [14]. Conversely, some elements are nonessential (e.g., cadmium, mercury, and lead), toxic, and harmful for physiological functions in bacteria at all concentrations, including at sub-chronic doses [15]. According to Chandrangsu et al. [16], the genetic features of the microbiomes could be significantly modified due to the effects of elevated concentrations of heavy metals and/or other pollutants in the environment. Long-term exposure to high chemical concentrations may cause bacteria to become resistant by developing new defense mechanisms against the stress caused by harmful substances. Thus, the presence of metal-resistant bacteria can become bioindicators of pollution events due to their microbial adaptive strategies [17].

To date, our current knowledge of heavy metal-tolerant bacteria isolated from Arctic and Antarctic environments remains scarce and fragmentary. Studies have mainly addressed heavy metal tolerance in bacteria from soil [18,19,20], marine biota [21,22], marine environments [23,24,25,26], and riverine systems [27,28], whereas few investigations have focused on bacteria from polar lakes [29]. Therefore, there are still many gaps to fill in our knowledge of the interactions between microorganisms and elements in extremely cold environments, and especially lacustrine systems.

The goal of this study was to isolate bacteria with potential for bioremediation applications. This was achieved by (i) enriching and isolating microorganisms from lacustrine sediment with tolerance for heavy metals under cold conditions, (ii) identifying the bacterial isolates, and (iii) assessing their metal biosequestration capability.

## 2. Materials and Methods

### 2.1. Sampling Activities

Sampling activities were carried out in eleven shallow lakes lying in the Arctic (four lakes in Ny-Ålesund, Svalbard Islands, Arctic Norway; coordinates 78°55′ N–11°56′ E) and Antarctica (seven lakes between Deception Island, coordinates 62°34′35″ S–60°54′14″ W, and Livingston Island, coordinates 62°57′ S–60°38′ W, in the South Shetland Islands). In the Arctic, sampling was performed between August 5th and 18th 2021 from lakes surrounding the Ny-Ålesund research village, as follows: Lake Solvannet (L1), Lake Glacier (L2), Lake Knudsenheia (L3), and Lake Storvatnet (L4). In Antarctica, sampling was performed between 25 January and 1 February 2022 from the following lakes: Lake Argentina (LA) and Lake Sofia (LS) (both in Livingston Island), Lake Ballaneros (LB), Lake Crater (LC), Lake Extremadura (LE), Lake Telefon (LT), and Lake Zapatilla (LZ) (Deception Island) (Table 1). The lakes Solvannet, Storvatnet, Argentina, and Crater were selected for sampling as impacted lakes because of their location (e.g., near the scientific bases) and their characteristics (e.g., presence of animals).

Trace Fe concentrations in the analyzed lakes ranged between 3.3 and 10 µg L^−1^, except for brackish lakes, where concentrations were approximately 300 µg L^−1^. A similar situation was found for Cu, which ranged from 0.5 to 3.9 µg L^−1^, except in brackish lakes, where values ranged between 291 and 601 µg L^−1^. Hg concentrations ranged between 0.2 and 1 µg L^−1^.

At each lake, water and sediment samples (depth 30–60 cm) were aseptically collected. Water (6 L) was collected in pre-sterilized polycarbonate containers. Sediments (first 10 cm; 200 g) were collected using a pre-sterilized metal scoop and sterile plastic containers. Samples were maintained at 4 °C during transportation to the laboratories of the Research Bases for preliminary processing. The main physical–chemical parameters (O_2_, pH, temperature, and conductivity) were recorded.

### 2.2. Setup of Enrichment Cultures

Enrichment cultures were set up by using 1 g of wet lake sediment to inoculate 75 mL of filter-sterilized lake water. Cultures were enriched with individual heavy metal (HM) salts, as follows: FeSO_4_ (Fe), CuSO_4_ (Cu), and HgCl_2_ (Hg) (Sigma, Burlington, MA, USA). Metal salt solutions were prepared in filter-sterilized 1X phosphate-buffered saline (PBS), as reported by Selvin et al. [30]. Fe and Cu were added at a final concentration of 1000 mg L^−1^, whereas Hg was added at a final concentration of 100 mg L^−1^. All enrichments were incubated aerobically at 4 °C for two months [27,28].

### 2.3. Isolation of HM-Tolerant Bacteria

Aliquots (100 μL) of each enrichment culture were spread-plated in duplicate on HM-amended nutrient agar (NA; Difco, Milan, Italy) using the same HM concentrations as in the enrichment setup (i.e., 1000 mg L^−1^ for Fe and Cu and 100 mg L^−1^ for Hg). Inoculated plates were incubated at 4 °C for 30 days. Bacterial colonies grown on HM-amended agar plates were randomly selected, picked, and subcultured on NA under the same conditions. HM-tolerant bacterial isolates were routinely grown on NA and maintained at 4 °C.

### 2.4. Bacterial Growth at Increasing HM Concentrations

A microtiter plate-based culture assay was set up to screen bacterial isolates for growth at increasing concentrations (up to 5000 mg L^−1^ for Fe and Cu, and up to 500 mg L^−1^ for Hg) of the same HM by amending the enrichment of the original culture. A modified version of the protocol described by Blasi et al. [31] was applied. Briefly, starting bacterial cultures were prepared by growing each bacterial strain in a microtiter plate well, as follows: Each well was filled with 250 µL of nutrient broth (NB) in duplicate. The plates were then incubated at 4 °C for 15 days. Bacterial growth was assessed by measuring the optical density of the cultures over time at 605 nm (OD605), using a microplate reader (Adsorbance 96 spectrophotometer, Byonoy, Hamburg, Germany). The recorded values were processed using the Absorbance 96 software version 1.2. Wells containing NB without bacterial inoculation were used as blanks for the OD readings. After incubation, 25 µL of each strain culture were transferred, in duplicate, into 225 µL of 10% NB amended with the HMs at the following concentrations: 2500 and 5000 mg L^−1^ for Fe and Cu, and 250 and 500 mg L^−1^ for Hg. The plates were then incubated at 4 °C for 15 days. Growth was assessed daily as described above. Wells containing 10% HM-amended NB (without bacterial inoculation) were used as negative controls.

### 2.5. Screening for Multitolerance

Bacterial strains capable of growing in the presence of high concentrations of HMs were further assayed for tolerance to HMs different from those used in the enrichment cultures. Selected strains were streaked on NA amended with HMs and incubated for 30 days at 4 °C. The concentrations used were 1000, 2500, and 5000 mg L^−1^ for Fe and Cu, and 100 mg L^−1^ and 250 mg L^−1^ for Hg [28].

### 2.6. Sequestration Rate of Heavy Metals

To test the HM sequestration rate, selected bacterial strains were inoculated in NB amended with 190 mg L^−1^ of mercury or 2250 mg L^−1^ of iron. To monitor abiotic losses, two negative controls were prepared and treated as the inoculated cultures: NB (i.e., without bacterial inoculation or metal addition) and NB*plus*HM (i.e., without bacterial inoculation but with the addition of 190 mg L^−1^ for Hg or 2250 mg L^−1^ for iron). After incubation for 30 days at 4 °C, the samples were centrifuged at 8000 rpm for 20 min at 4 °C to separate the liquid phase from the pellet. The samples for analysis were diluted (1:1000) in a solution of one liter containing 0.1% of Triton X, 1% of nitric acid, and 0.1% of EDTA, and then analyzed by Inductively Coupled Plasma Optical Emission spectroscopy (ICP-OES; characteristics of the instrument in [32]), according to Turetta et al. [33].

### 2.7. Identification of Bacterial Isolates

Bacterial isolates were identified by 16S rRNA gene sequencing. Briefly, single bacterial colonies were lysed by heating (95 °C for 10 min). Amplification of the 16S rRNA gene was performed with a thermocycler (Mastercycler GeneAmp PCR-System 9700, Applied Biosystems, Waltham, MA, USA) using the bacteria-specific primers 27F (5′–AGAGTTTGATC(AC)TGGCTCAG–3′) and 1492R (5′–TACGGYTACCTTGTTACGAC-3′). The reaction mixtures were assembled at 0 °C. The composition of the reaction mixture was (final volume: 25 μL) 2 μL DNA, 1 μL of each of the two primers (10 μM), 5 μL of reaction buffer 10×, 0.25 μL of Taq polymerase My Taq PRIME (5 U μL^−1^), and sterile Milli-Q water. Negative controls for DNA extraction and PCR setup (reaction mixture without a DNA template) were also used in every PCR run. The PCR program used was (1) 95 °C for 1 min; (2) 35 cycles at 95 °C for 30 s, 52 °C for 30 s, and 72 °C for 30 s; and (3) 72 °C for 10 min. Quality of the DNA was checked by running 2 μL of each sample on 1% agarose gel (*w*/*v*) (Bioline, Meridian Bioscience, Menphis, TN, USA) in TAE buffer 1X (0.04 M Tris-acetate, 0.02 M acetic acid, 0.001 M EDTA) (Fermentas, Waltham, MA, USA) containing SYBR™ Safe (1 µL/25mL final concentration) (Invitrogen, Waltham, MA, USA). Amplified products were sequenced by Eurofins Europe (Konstanz, Germany). The FinchTV (version 1.4) program was used to read and correct the manually obtained sequences. Next relatives of isolates were determined by comparing sequences in the NCBI GenBank and the EMBL databases using BLAST and the “Seqmatch” and “Classifier” programs of the Ribosomal Database Project II (https://blast.ncbi.nlm.nih.gov/Blast.cgi?PROGRAM=blastn&BLAST_SPEC=GeoBlast&PAGE_TYPE=BlastSearch, accessed on 5 February 2025) [34]. Sequences were further aligned using the program Clustal W (webpage link https://www.genome.jp/tools-bin/clustalw, accessed on 5 February 2025) to the most similar orthologous sequences retrieved from databases.

### 2.8. Statistical Analysis

Results obtained for microbial growth in the enrichment cultures and metal concentrations in the analyzed samples were used to perform linear regression and Spearman correlation, using R software version 4.4.2 with the base R package stats version 4.4.2.

## 3. Results

### 3.1. Bacterial Isolation

Overall, 58 and 43 bacterial strains were isolated from Arctic and Antarctic lakes, respectively. In particular, 90 were from Fe enrichments (1000 mg L^−1^); 50 and 40 were from Arctic and Antarctic lakes, respectively, i.e., 16 from Lake Knudsenheia, 13 from Lakes Solvannet, 11 from Lake Glacier, and 10 from Lake Storvatnet in the Arctic, and 13 from Lake Telefon, 13 from Lake Ballaneros, 7 from Lake Zapatilla, 4 from Lake Extremadura, and 3 from Lake Argentina in Antarctica. Additionally, eight were from the Hg enrichments (100 mg L^−1^), all from the Arctic Lake Storvatnet, and three were from Cu enrichments (1000 mg L^−1^), all from the Antarctic Lake Ballaneros.

### 3.2. Bacterial Growth at Increasing HM Concentrations

As stated above, all isolates were then tested for growth at metal concentrations higher than those used for the enrichments, i.e., Fe and Cu up to 5000 mg L^−1^ and Hg up to 500 mg L^−1^. None of the Cu- and Hg-tolerant strains were able to grow at higher concentrations of Cu and Hg. Conversely, 10 and 15 isolates from Antarctic and Arctic lake water, respectively, enriched with Fe, were able to grow in the presence of up to 5000 mg L^−1^ of Fe, even if, in some cases, bacterial growth was weak (Table 2).

Fe-tolerant bacterial isolates were successfully identified by 16S rRNA gene sequencing and their phylogenetic affiliation is shown in Supplementary Table S1. Sequences of six isolates were not obtained due to poor amplification and/or bad sequencing quality.

The majority of identified Fe-tolerant bacterial isolates from Arctic lake sediments were affiliated with the genus *Pseudomonas* (within Gammaproteobacteria). Additionally, two strains belonged to the genus *Janthinobacterium* (within Betaproteobacteria), while a single strain was affiliated with the genus *Carnobacterium* (within Bacillota). Unfortunately, identification was not successful for four strains (i.e., S2A-1, S2A-4, S2A-5, and S4A-2).

Identified Fe-tolerant bacterial isolates from Antarctic lake sediment were mainly affiliated with the phylum Actinomycetota, within the genera *Subtercola*, *Pseudoarthrobacter,* and *Arthrobacter*. Two other isolates were affiliated with the genus *Janthinobacterium* (within Betaproteobacteria). Unfortunately, the phylogenetic affiliation could not be determined for two strains (ABA-4 and AZA-3). Antarctic isolates are part of the Italian Collection of Antarctic Bacteria of the National Antarctic Museum (CIBAN-MNA), kept at the University of Messina (voucher codes MNA-CIBAN-2266 to MNA-CIBAN-2275; Supplementary Table S1).

### 3.3. Screening for Multitolerance

Only four bacterial isolates from those that tolerated up to 5000 mg L^−1^ of Fe grew (even if weakly) at higher concentrations of Cu (Table 3).

In detail, *Pseudomonas* spp. S2A-8 and S3A-11, from the Arctic, tolerated up to 1000 mg L^−1^ of this metal. Among the Antarctic isolates, the strain ABA-4 (not identified) tolerated up to 5000 mg L^−1^ of Cu, while *Subtercola* sp. AZA-8 grew at a Cu concentration of 2500 mg L^−1^. A total of 16 bacterial strains (out of 25) tolerated Hg up to 250 mg L^−1^, with better growth generally shown by Antarctic rather than Arctic isolates. Finally, only three strains tolerated Hg up to 500 mg L^−1^; these were *Pseudomonas* sp. S1A-14 and S2A-5 (not identified) from Arctic lakes, and *Subtercola* sp. AZA-8 from Antarctica. Six isolates did not tolerate Cu and Hg at the tested concentrations.

### 3.4. Statistical Results

The statistical analyses carried out on obtained data did not show significant results, except for the Spearman table showing significative positive correlations among the number of colonies counted on Fe enrichment cultures and metal concentrations in the analyzed lakes (Supplementary Figure S1). Fe, Cu, and Hg were also positively correlated.

### 3.5. Sequestration Rate of Heavy Metals

**Figure 1:**
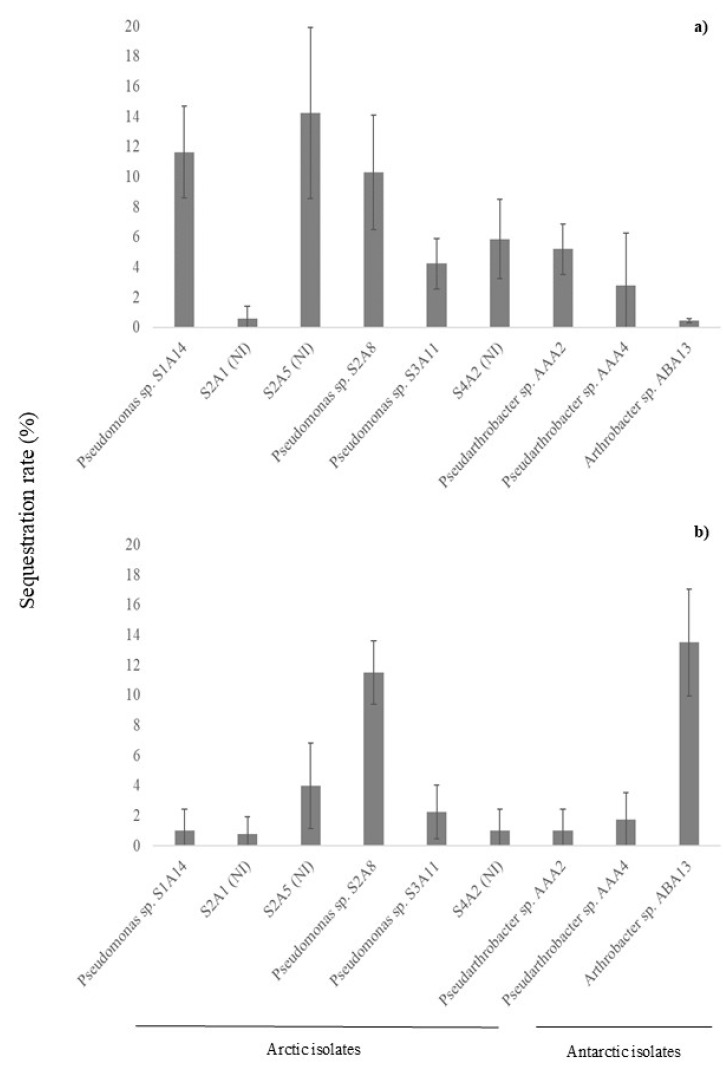
Sequestration of (**a**) mercury and (**b**) iron by Arctic and Antarctic bacteria.

Based on the results reported above, three and six isolates from Antarctica and the Arctic, respectively, were selected to carry out further evaluation of their sequestration rates for Fe and Hg. Even if some strains tolerated Cu, their growth in the presence of this metal was too weak to proceed with further tests. Isolates from the Arctic were *Pseudomonas* spp. S1A-14, S2A-8, and S3A-11; those not identified included S2A-1, S2A-5, and S4A-2. Antarctic isolates were *Pseudarthrobacter* spp. AAA-2 and AAA-4 and *Arthrobacter* sp. ABA-13. As shown in Figure 1a, both Arctic and Antarctic isolates exhibit a sequestration rate of Hg ranging from below 6% to 14.2%. No abiotic losses were observed in the control samples. The Arctic strain S2A-5 was the most promising isolate as it sequestered approximately 14.2% of the metal, followed by *Pseudomonas* spp. S1A-14 and S2A-8 (11.7 and 10.3%, respectively, both from Arctic lakes). For Fe sequestration (Figure 1b), the most promising strain was *Arthrobacter* sp. ABA-13 (13.4%) from Antarctica, followed by the Arctic *Pseudomonas* sp. S2A-8 (11.9%). The remaining bacterial isolate strains showed sequestration rates below 4%.

## 4. Discussion

There is increasing interest in studying heavy metal tolerant-microorganisms from natural environments because of their high potential for industrial applications and their applicability in situ in bioremediation. To date, there is a lack of knowledge on the existence of heavy metal-tolerant cold-adapted bacteria (both psychrophilic and psychrotolerant), especially in lacustrine habitats in the Arctic and Antarctica. In this study, 58 heavy metal-tolerant bacterial isolates (to copper, iron, and mercury) were tested at 4 °C. Tests at higher concentrations of these metals showed that Fe was better tolerated than Cu and Hg. This finding is in line with previous studies reporting Fe resistance as a common trait in environmental bacteria, even in cold regions, likely due to its essential role in microbial metabolism and availability in sediment environments [14,35]. In fact, iron is crucial for bacterial growth and survival, as it is a common co-factor in essential enzymes [36]. Natural lithogenic/geogenic sources of heavy metals resulting in naturally induced resistance should also be considered [37]. For instance, the profiles of the microbial resistome (genes related to antibiotic, biocide, and heavy metal resistances) were evaluated using a metagenomics approach in Antarctic areas with and without any anthropogenic impact [38]. While all resistance genes were found in impacted sites, only genes for heavy metal tolerance were retrieved from non-impacted regions.

To the best of our knowledge, the heavy metal-tolerant isolates in this study were closely related to taxa that have never been reported in the scientific literature as metal-resistant or -tolerant. On the contrary, some of them have been listed in two papers in relation to bacterial diversity in snow in Ny-Ålesund and antibiotic resistance [39,40]. While their presence in microbial diversity studies is expected, their antibiotic resistance capacity indicates an ability to adapt to environmental stresses. Given the well-known relationship between metal and antibiotic resistance, this reinforces the relevance of our study in characterizing these microorganisms in metal-rich environments.

A distinctive feature of the lake sediment we analyzed was the prevalence of Proteobacteria (mainly Gammaproteobacteria in the genus *Pseudomonas*) and Actinomycetota (genera *Arthrobacter*, *Pseudoarthrobacter,* and *Subtercola*) in the Arctic and Antarctic Fe-tolerant bacterial isolates, respectively. Actinomycetota could possess a slight advantage in iron sequestration due to their robust cell wall structures, which can facilitate enhanced metal binding. Conversely, Gammaproteobacteria may prioritize resistance mechanisms such as efflux systems over sequestration [41]. The genus *Janthinobacterium* (among Betaproteobacteria) was common to both regions, whereas a single *Carnobacterium* representative (among Firmicutes) was isolated from an Arctic lake. *Pseudomonas* and *Janthinobacterium* were among the prevalent genera harboring resistome genes in a study carried out in marine sediments at Deception Island [26]. *Pseudomonas* species show extraordinary resilience, thus surviving and becoming dominant in environments characterized by extreme conditions and limited nutrient availability. *Pseudomonas* members are known for their ability to reduce a wide variety of metals [36,37,42,43,44]. The resistome profile of an Antarctic *Pseudomonas* against multiple antibiotics, biocides and metals was analyzed recently [45]. *Janthinobacterium* was reported to not tolerate heavy metals, such as Ag, Cu, Hg, Pb, and Ni [46]. In a study by Yin et al. [47], the relative abundance of *Janthinobacterium* decreased in highly contaminated samples. The tolerance of *Carnobacterium* members to heavy metals, such as Cd and Sb, has been assessed [48], also in cold environments [44]. Finally, Actinomycetota are well known for their metabolic versatility and bioactive properties, which make them particularly attractive for application in bioremediation fields [49]. Heavy metal tolerance in cold-adapted *Arthrobacter* members has been frequently reported (e.g., [19,39,50,51]). Comprehensive genome sequencing of the psychrotolerant *Arthrobacter* sp. PAMC25284 from Antarctic revealed the presence of genes involved in copper resistance [52]. *Pseudoarthrobacter* members, from warmer areas, have seldom been reported as heavy metal-tolerant [53,54]. Finally, to the best of our knowledge, we report for the first time the ability of the genus *Subtercola* to grow in the presence of heavy metals, as its ability to grow even weakly at higher concentrations is remarkable. In this study, only the Arctic *Pseudomonas* sp. S1A-14 and S2A-5 (not identified) and the Antarctic *Subtercola* sp. AZA-8 were able to grow at 500 mg L^−1^ of Hg. In general, mercury was the least-tolerated metal, with most Arctic and Antarctic isolates growing only up to 250 mg L^−1^ of Hg.

Overall, this assessment of metal sequestration revealed limited capacities in the bacterial isolates tested. With respect to iron, the Antarctic *Arthrobacter* sp. ABA-13 and the Arctic *Pseudomonas* sp. S2A-8 achieved the highest rates, at about 13%. Similarly, 10–14% of Hg was removed from the culture medium, with an unidentified strain of S2A5 together with *Pseudomonas* sp. S2A-8 and *Pseudomonas* sp. S1A-14 (all from Arctic lakes) as the most promising isolates. These findings suggest that even if polar bacteria can tolerate heavy metals, their sequestration efficiency remains modest, potentially limiting their direct application in bioremediation [24]. Nevertheless, the genetic determinants of resistance in these taxa could provide valuable insights when developing biotechnological applications for heavy metal bioremediation in cold areas. Future work should focus on characterizing resistance genes and their regulatory pathways and exploring co-culture strategies to enhance sequestration rates. Therefore, observations from this study constitute a baseline for further analysis aimed at better elucidating the metabolizing capabilities of bacteria from polar lacustrine sediments.

## Figures and Tables

**Table 1:** Arctic and Antarctic lakes investigated in this study. The main features of the sampled lakes are indicated in the last column: A/A = lake influenced by animals and humans; B = brackish lake; G = glacial lake.

Polar Area	Lake	Lake ID	Coordinates	Physical–Chemical Parameters				
				Temp.	pH	O_2_	Cond.	Main
				(°C)		(%)	(uS/cm)	Features
Ny-Ålesund (Arctic Norway)	Solvannet	L1	78°55.552′ N–11°56.327′ E	6.2	8.17	98.0	395	A/A
	Glacier	L2	78°55.044′ N–11°47.442′ E	8.7	7.66	98.2	151	G
	Knudsenheia	L3	78°56.680′ N–11°51.579′ E	8.8	8.36	105.3	2620	B
	Storvatnet	L4	78°55.453′ N–11°52.728′ E	7.9	8.07	103.2	243	A/A
Livingston Island (Antarctica)	Sofia	LS-1	62°40′12.19″ S–60°23′17.90″ W	0.3	5.2	69.4	26.5	G
	Argentina	LA	62°40′22.39″ S–60°24′18.12″ W	1.4	5.7	68.1	64.6	A/A
Deception Island (Antarctica)	Crater	LC	62°59′00.68″ S–60°40′20.41″ W	3.7	5.5	86.0	6.64	A/A
	Zapatilla	LZ	62°59′00.24″ S–60°40′29.07″ W	6.8	5.6	76.5	55.6	Water supply
	Extremadura	LE	62°55′12.2″ S–60°39′47.0″ W	4.1	7.2	94.8	448	B
	Telefon	LT	62°55′39.9″ S–60°41′21.3″ W	5.4	6	-	507	B
	Ballaneros	LB	62°58′51.1″ S–60°34′27.1″ W	4.1	4.7	67.9	480	B

**Table 2:** Arctic and Antarctic isolates growing at increasing concentrations of Fe. (+), weak growth; +, normal growth; ++, conspicuous growth.

Lake of Origin	Isolate	Fe (mg L^−1^)	
		2500	5000
**Arctic Lakes**			
**Lake Solvannet (L1)**	*Pseudomonas* sp. S1A-14	+	+
**Lake Glacier (L2)**	S2A-1 (not identified)	+	+
	S2A-2 (not identified)	+	+
	*Carnobacterium* sp. S2A-4	+	(+)
	S2A-5 (not identified)	+	(+)
	*Janthinobacterium* sp. S2A-6	(+)	(+)
	*Janthinobacterium* sp. S2A-7	+	+
	*Pseudomonas* sp. S2A-8	++	+
**Lake Knudsenheia (L3)**	*Pseudomonas* sp. S3A-2	+	+
	*Pseudomonas* sp. S3A-11	+	+
**Lake Storvatnet (L4)**	*Pseudomonas* sp. S4A-1	(+)	(+)
	S4A-2 (not identified)	+	+
	*Pseudomonas* sp. S4A-4	+	+
	*Pseudomonas* sp. S4A-7	+	(+)
	*Pseudomonas* sp. S4A-10	+	+
**Antarctic Lakes**			
**Lake Ballaneros (LB)**	ABA-4 (not identified)	(+)	(+)
	*Arthrobacter* sp. ABA-13	(+)	(+)
**Lake Argentina (LA)**	*Pseudarthrobacter* sp. AAA-2	++	+
	*Pseudarthrobacter* sp. AAA-4	+	(+)
**Lake Zapatilla (LZ)**	*Pseudarthrobacter* sp. AZA-2	++	+
	AZA-3 (not identified)	++	(+)
	*Pseudarthrobacter* sp. AZA-4	++	+
	*Subtercola* sp. AZA-8	++	(+)
	*Subtercola* sp. AZA-9	+	(+)
**Lake Telefon (LT)**	*Arthrobacter* sp. ATA-13	(+)	(+)

**Table 3:** Arctic and Antarctic isolates tolerating more than one heavy metal salt. (+), weak growth; +, normal growth; −, no growth.

Polar Area	ID Strain	Cu (mg L^−1^)			Hg (mg L^−1^)		
		1000	2500	5000	100	250	500
Arctic	*Pseudomonas* sp. S1A-14	−	−	−	+	+	(+)
	S2A-1 (not identified)	−	−	−	−	−	−
	S2A-2 (not identified)	−	−	−	−	−	−
	*Carnobacterium* sp. S2A-4	−	−	−	−	−	−
	S2A-5 (not identified)	−	−	−	+	(+)	(+)
	*Janthinobacterium* sp. S2A-6	−	−	−	−	−	−
	*Janthinobacterium* sp. S2A-7	−	−	−	+	+	−
	*Pseudomonas* sp. S2A-8	(+)	−	−	+	(+)	−
	*Pseudomonas* sp. S3A-2	−	−	−	+	(+)	−
	*Pseudomonas* sp. S3A-11	(+)	−	−	+	+	−
	*Pseudomonas* sp. S4A-1	−	−	−	+	+	−
	S4A-2 (not identified)	−	−	−	+	+	−
	*Pseudomonas* sp. S4A-4	−	−	−	+	+	−
	*Pseudomonas* sp. S4A-7	−	−	−	+	+	−
	*Pseudomonas* sp. S4A-10	−	−	−	+	+	−
Antarctica	ABA-4 (not identified)	(+)	(+)	(+)	(+)	(+)	−
	*Arthrobacter* sp. ABA-13	−	−	−	+	+	−
	*Pseudarthrobacter* sp. AAA-2	−	−	−	+	+	−
	*Pseudarthrobacter* sp. AAA-4	−	−	−	+	(+)	−
	*Pseudarthrobacter* sp. AZA-2	−	−	−	+	(+)	−
	AZA-3 (not identified)	−	−	−	+	(+)	−
	*Pseudarthrobacter* sp. AZA-4	−	−	−	+	(+)	−
	*Subtercola* sp. AZA-8	(+)	(+)	−	(+)	(+)	(+)
	*Subtercola* sp. AZA-9	−	−	−	−	−	−
	*Arthrobacter* sp. ATA-13	−	−	−	−	−	−

## Data Availability

Nucleotide sequences have been deposited in the GenBank database under the accession nos. PQ686992 and PQ686999-PQ687016 (see Supplementary Table S1 for details). The data that support the findings of this study are available from the authors upon request.

## Supplementary Materials

The following supporting information can be downloaded at https://www.mdpi.com/article/10.3390/microorganisms13020389/s1: Table S1: 16S rRNA gene sequence affiliation to their closest phylogenetic neighbors of Arctic and Antarctic isolates. Figure S1: Spearman correlation table for Fe, Cu, and Hg concentrations in all analyzed lakes, along with the number of bacterial colonies grown in metal-enriched cultures. The table includes correlation coefficients and *p*-values, while the visualization highlights statistically significant correlations (*p* < 0.05) using ellipses, with numbers indicating the strength and direction of the correlation.
