# Supplementary material for: Enrichment, Isolation and Characterization of Heavy Metal-Tolerant Bacteria from Polar Lacustrine Sediments
Source: Microorganisms. 2025 Feb 10;13(2):389. doi: 10.3390/microorganisms13020389 (PMC11858113; doi:10.3390/microorganisms13020389)
Supplement: Supplementary file 1 [file microorganisms-13-00389-s001.zip › microorganisms-3444744-supplementary.pdf]

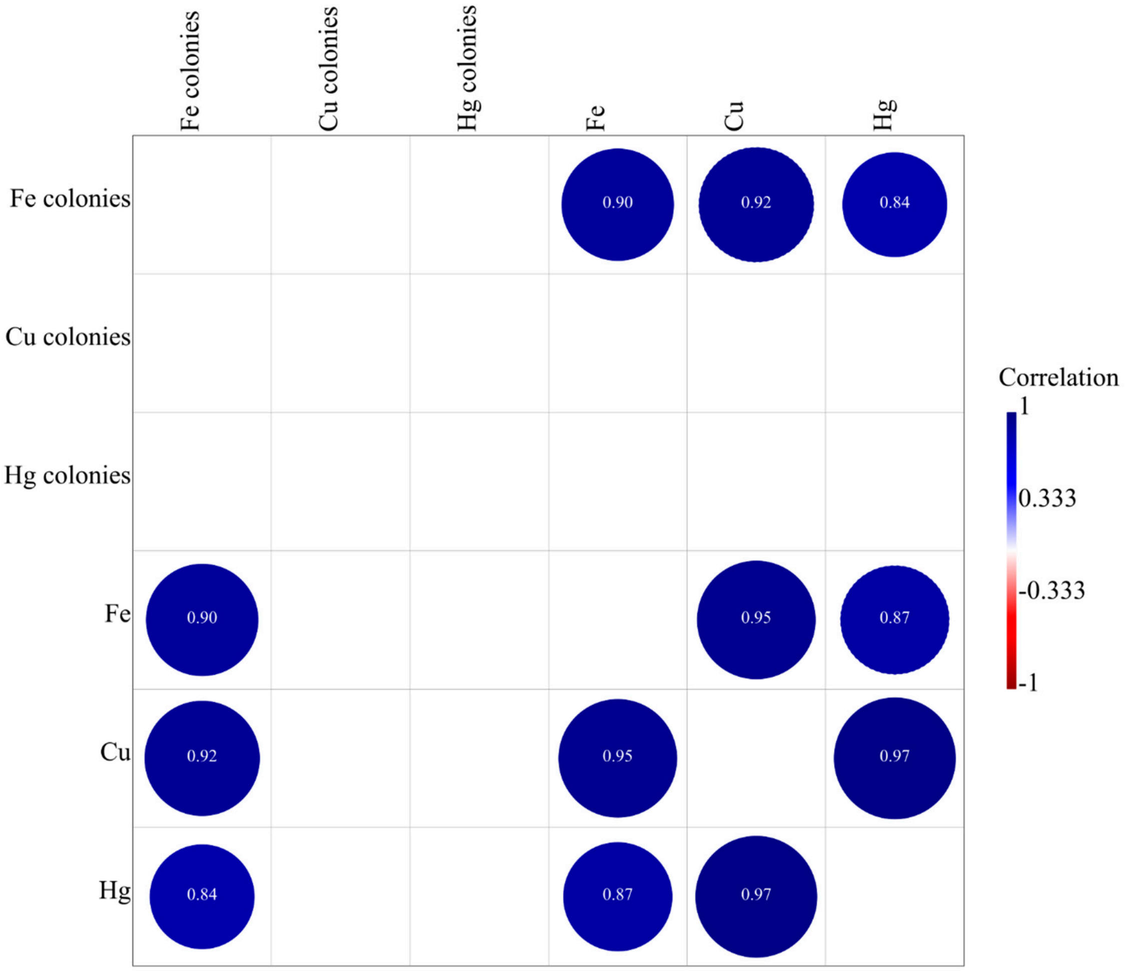

Figure S1: Spearman correlation table for Fe, Cu, and Hg concentrations in all analysed lakes, along with the number of bacterial colonies grown in metal-enriched cultures. The table includes correlation coefficients and p-values, while the visualization highlights statistically significant correlations ($p < 0.05$) using ellipses, with numbers indicating the strength and direction of the correlation.

Supplementary Table S1. 16S rRNA gene sequence affiliation to their closest phylogenetic neighbours of Arctic and Antarctic isolates.

	Strain ID	Next relative by GenBank alignment (AN[a], organism)	Phylum or class	Accession Number	Hom[b] (%)	MNA voucher
Arctic isolates	S2A-4	KC213942, *Carnobacterium maltaromaticum* strain S_S_MRS_1	*Firmicutes*	PQ686992	100.00	
	S4A-7	MW633299, *Janthinobacterium* sp. strain F2TT2	*Betaproteobacteria*	PQ687006	88.93	
	S2A-6	JF312926, *Janthinobacterium* sp. HC1-14	*Betaproteobacteria*	PQ687000	99.90	
	S1A-14	KR233795, *Pseudomonas antarctica* strain IHB B 6450	*Gammaproteobacteria*	PQ686999	100.00	
	S4A-4	KX186936, *Pseudomonas antarctica* culture DSM:15318	*Gammaproteobacteria*	PQ687005	99.62	
	S4A-10	MG576168, *Pseudomonas antarctica* strain KBL15	*Gammaproteobacteria*	PQ687007	97.91	
	S2A-7	MN826583, *Pseudomonas extremaustralis* strain cqsV14	*Gammaproteobacteria*	PQ687001	100.00	
	S2A-8	MN826583, *Pseudomonas extremaustralis* strain cqsV14	*Gammaproteobacteria*	PQ687002	99.80	
	S3A-2	MK590240, *Pseudomonas* sp. strain SYMB27	*Gammaproteobacteria*	PQ687003	99.81	
	S3A-11	MK590240, *Pseudomonas* sp. strain SYMB27	*Gammaproteobacteria*	PQ687004	99.73	
	S4A-1	MK590240, *Pseudomonas* sp. strain SYMB27	*Gammaproteobacteria*	PQ687008	99.54	
	S2A-1	Not identified				
	S2A-4	Not identified				
	S2A-5	Not identified				
	S4A-2	Not identified				
Antarctic isolates	AZA-8	MK248095, *Subtercola frigoramans* strain RGC-4	*Actinomycetota*	PQ687015	97.62	CIBAN-MNA-2266
	AZA-9	MK248095, *Subtercola frigoramans* strain RGC-4	*Actinomycetota*	PQ687016	99.52	CIBAN-MNA-2267
	ATA-13	AB920568, *Arthrobacter alpinus* strain: S6-3	*Actinomycetota*	PQ687012	99.12	CIBAN-MNA-2268
	ABA-13	MW928757, *Arthrobacter livingstonensis* strain NJ-QEDFT-5-B	*Actinomycetota*	PQ687011	99.55	CIBAN-MNA-2269
	AAA-2	KF528714, *Pseudarthrobacter scleromae* strain PAMC 25156	*Actinomycetota*	PQ687009	99.90	CIBAN-MNA-2270
	AAA-4	KF528714, *Pseudarthrobacter scleromae* strain PAMC 25156	*Actinomycetota*	PQ687010	99.82	CIBAN-MNA-2271
	AZA-2	OP379315, *Janthinobacterium svalbardensis* strain RS076	*Betaproteobacteria*	PQ687013	99.90	CIBAN-MNA-2272
	AZA-4	MN524134, *Janthinobacterium* sp. strain SNU WT3	*Betaproteobacteria*	PQ687014	99.52	CIBAN-MNA-2273
	ABA-4	Not identified				CIBAN-MNA-2274
	AZA-3	Not identified				CIBAN-MNA-2275
